# Environmental factors in offspring of parents with mood disorders and their role in parent–child transmission: findings from a 14-year prospective high-risk study

**DOI:** 10.1186/s40345-022-00257-5

**Published:** 2022-04-06

**Authors:** Flore Moulin, Mehdi Gholam, Marie-Pierre F. Strippoli, Enrique Castelao, Kathleen R. Merikangas, Emma K. Stapp, Pierre Marquet, Jean-Michel Aubry, Kerstin J. Plessen, Francesca Di Giacomo, Jennifer Glaus, Giorgio Pistis, Benjamin Lavigne, Julien Elowe, Setareh Ranjbar, Martin Preisig, Caroline L. Vandeleur

**Affiliations:** 1 Department of Psychiatry, Centre for Research in Psychiatric Epidemiology and Psychopathology, Lausanne University Hospital and University of Lausanne, Route de Cery 25, Prilly, Switzerland; 2 INSERM U 1219, Bordeaux Population Health Research Center, Bordeaux, France; 3 Genetic Epidemiology Research Branch, Intramural Research Program, National Institute of Mental Health, Bethesda, MD USA; 4 Department of Psychiatry, Lausanne University Hospital and University of Lausanne, Lausanne, Switzerland; 5 International Research Unit in Neurodevelopment and Child Psychiatry, Laval University, Quebec, Canada; 6 Department of Psychiatry, University Hospital of Geneva, Geneva, Switzerland; 7 Division of Child and Adolescent Psychiatry, Department of Psychiatry, Lausanne University Hospital and University of Lausanne, Lausanne, Switzerland; 8 Department of Psychiatry, Lausanne University Hospital and University of Lausanne, North Sector, Canton of Vaud, Yverdon, Switzerland; 9 Department of Psychiatry, Lausanne University Hospital and University of Lausanne, West Sector, Canton of Vaud, Prangins, Switzerland; 10 Department of Psychiatry, Lausanne University Hospital and University of Lausanne, North and West Sectors, Canton of Vaud, Yverdon and Prangins, Switzerland

**Keywords:** Offspring of bipolar parents, Offspring of depressed parents, Family environment, Trauma, Parental separation

## Abstract

**Background:**

The factors involved in the transmission of mood disorders are only partially elucidated. Aside from genes, the family environment might play a crucial role in parent–child transmission. Our goals were to (1) assess the associations of parental bipolar disorder (BPD) and Major Depressive Disorder (MDD) with individual or shared family environmental factors, including traumatic events in offspring, parental separation, family cohesion and parental attitudes; and 2) test whether these factors were mediators of the association between exposure to parental mood disorders and the onset of these disorders in offspring.

**Methods:**

The sample stems from an ongoing family high-risk study of mood disorders conducted in the French-speaking part of Switzerland. Given the strong impact of the age of onset of parental disorders on their transmission to children, parental disorders were dichotomized according to the onset (cut-off 21 years). Probands with early-onset (n = 30) and later-onset BPD (n = 51), early-onset (n = 21) and later-onset MDD (n = 47) and controls (n = 65), along with their spouses (n = 193) and offspring (n = 388; < 18 years on study inclusion), were assessed over a mean follow-up duration of 14 years (s.d: 4.6). The environmental measures were based on reports by offspring collected before the onset of their first mood episode.

**Results:**

Offspring of probands with later-onset BPD and offspring of probands with both early-onset and later-onset MDD reported traumatic events more frequently than comparison offspring, whereas exposure to parental separation was more frequent in all groups of high-risk offspring. Moreover, several familial environment scores including parenting attitudes differed between offspring of probands with BPD and comparison offspring. However, none of these factors were mediators of the parent–child transmission of BPD. Among the environmental factors, traumatic events were shown to be modest mediators of the transmission of early-onset MDD.

**Conclusions:**

Our data do not support the implication of the assessed environmental factors in the parent–child transmission of BPD. In contrast to BPD, traumatic events partially mediate the parent–child transmission of early-onset MDD, which has important implications for intervention and prevention. Early therapeutic efforts in offspring exposed to these events are likely to reduce their deleterious impact on the risk of subsequent MDD.

## Introduction

Although mood disorders and particularly bipolar-I disorder have a strong familial component (Merikangas et al. 2014; Vandeleur et al. 2014), the factors involved in the parent–child transmission of these disorders are only partially elucidated, which impedes prevention (Stapp et al. 2020a). Indeed, the identification of modifiable risk and protective factors associated with the development of psychiatric disorders among offspring of affected parents is critical to enhance primary or secondary prevention (Stapp et al. 2020a). Aside from genetic transmission, the family environment might play a crucial role in the parent–offspring transmission of mood disorders (Beardslee et al. 2011; Menculini et al. 2020). Indeed, children growing up in the homes of parents with psychiatric disorders may be exposed to detrimental familial environments in terms of dysfunctional family dynamics, poor parental rearing and early adversity (Menculini et al. 2020; Johnson et al. 2001). Mostly cross-sectional research has consistently shown the familial environment of parents with bipolar disorders (BPD), or of youngsters affected by BPD themselves, to entail more interpersonal difficulties including lower cohesion and higher conflict, respectively, compared to families with no psychiatric disorders (review Stapp et al. 2020a). Recently, one prospective high-risk study showed parental BPD as well as other parental disorders to predict family impairment, cohesion gradually decreasing and conflict levels increasing from childhood across adolescence (Shalev et al. 2019). Poor parental rearing was associated with both internalizing and externalizing problems among offspring of affected parents (Iacono et al. 2017; Lau et al. 2018) and the community (Eun et al. 2018), whereas poor parent–child relationships associated with childhood maltreatment by age 11 predicted the onset of Major Depressive Disorders (MDD) among offspring from a large community study (Wilson et al. 2014). Furthermore, physical or sexual abuse in particular, which have frequently been reported in families of parents with mood disorders, have been shown to have lasting detrimental effects on offspring mental and physical health across the lifespan (Stapp et al. 2020b, reviews: Aas et al. 2016; Palmier-Claus et al. 2016), whereas the combination of a familial loading for psychiatric disorders across two generations and endured childhood adversity was associated with an earlier onset of BPD in a retrospective clinical study of adult outpatients with BPD (Post et al. 2016).

However, many studies on familial environmental factors were conducted once offspring had already started to develop psychopathology, which could have had an impact on the familial environment and influenced the child's assessment of this environment. Moreover, in most studies familial environmental factors were assessed using parental reports, which may have been influenced by the parental disorder. Therefore, there is a need of prospective studies that assess the family environment according to the offspring’s perspective (Backer et al. 2017), prior to the onset of their mood psychopathology. The few longitudinal studies on this topic (for an overview of longitudinal clinical high-risk studies studying the role of environmental factors in the transmission of mood disorders, see Table 1) suggested that higher perceived neglect from mothers (Doucette et al. 2016) or stressful life events (Kemner et al. 2015) in families with a parent with BPD, and adverse events including separation from parents in families with a parent with depression liability (Zimmermann et al. 2008), predicted the incidence of mood psychopathology in offspring. However, these studies generally focused on one or only a few factors at a time, whereas it is likely that a series of environmental factors simultaneously exert their influence on the development of offspring psychopathology (Barker et al. 2012). Moreover, many existing studies have not taken parental comorbidity or spouse psychopathology into account (Stapp et al. 2020a), which may also affect the familial environment and increase the risk of developing psychopathology. We could not identify any recent studies of offspring of parents with MDD recruited in clinical settings that studied the role of the family environment in parent–child transmission.

**Table 1:** Overview of longitudinal clinical high-risk studies studying the role of environmental risk factors in the parent–child transmission of mood disorders

Article, country, name of study	BPD parent type	Parent, type of control	Age range offspring	Diagnostic and environmental measures	Environmental factors	Offspring of parent with BPD		Control offspring		Main findings
						n (% female)	Mean age ± SD	n (% female)	Mean age ± SD	
Koenders et al. (2020), The Netherlands, “The Dutch Bipolar Offspring Study”	I, II	–	12–21	DSM-IV K-SADS-PL QFP CTQ	Childhood trauma Family functioning	102 (46)	16.0 ± 2.7	–	–	Among offspring of parents with BPD, emotional maltreatment (abuse and neglect) was significantly associated with mood disorder development. Due to very low variance on the physical trauma and sexual abuse scales, these were not incorporated in the analyses No association was found with the family functioning total score nor its subscales
Shalev et al. (2019), USA, “The Pittsburgh Bipolar Offspring Study”	I, II	w/o BPD dx or w/o any Psychiatric Dx (HC)	7–18	DSM-IV K-SADS-PL K-SADS-MRS K-SADS-P FACES II CBQ	Family functioning Family conflict	481 (50)	15.4 ± 2.6	*Offspring of parents w/o BPD dx* 162 (51.2) *Offspring of HC parents* 175 (52)	*Offspring of parents* w*/o BPD dx* 15.5 ± 2.4 *Offspring of HC parents* 15.5 ± 2.2	Families of parents with BPD and those of parents with non-BPD psychopathology showed lower cohesion and adaptability and higher conflict compared with HC families. There were no significant differences in cohesion and adaptability scores between families of parents with BPD and families of parents with non-BPD psychopathology In all 3 groups, parent-reported family conflict was significantly higher than child-reported conflict
Iacono et al. (2017), Quebec, Canada	I, II	w/o any psychiatric Dx, no lifetime mood Dx	4–21	SCID-I K-SADS-PL PDI CBCL TRF	Parenting practices (support, structure, control)	77 (NR)	8.4 ± 2.5	68 (NR)	12.4 ± 3.2	Parents with BPD showed impairment in parenting practices compared to controls in terms of less support, structure and control to their offspring in middle childhood. Low levels of structure mediated the relation between parental BPD and internalizing and externalizing difficulties during middle childhood. However, low parental control in middle childhood emerged as the strongest mediator of the relation between parental BPD and offspring psychopathology in late adolescence and early adulthood, in terms of substance misuse and depressive disorders among the offspring 12 years later
Kemner et al. (2015), The Netherlands, “The Dutch Bipolar Offspring Study”	I, II	–	12–21	DSM-IV K-SADS-PL LEDS TCI UCL Short-EMBU	Stressful life events Passive coping style Harm-avoidance temperament	140 (49)	16.0 ± 2.7	–	–	Among offspring of parents with BPD, stressful life events were a risk factor for the onset and recurrence of mood disorders. Passive coping style increased the risk of mood episode onset and recurrent episodes, but also altered the effect of life events on the onset of mood disorders by more than 10%, suggesting that having more passive reacting coping-style features enhanced the risk of mood episode onset. The impact of life-events was most pronounced in the early stages of mood disorders. Harm-avoidance temperament also increased the risk of subsequent mood episodes in offspring
Doucette et al. (2016), Canada, “The Canadian Flourish high-risk offspring cohort study”	I, II	–	16–23	DSM-IV K-SADS-PL CECA.Q EAS LEQ Hollingshead SES Scale Self-report measures of temperament and early adversities	Early childhood adversity Emotionality Exposure to parental BPD Stressful life events	233 (59.7)	16.6 ± 5.6	–	–	In offspring of parents with BPD, perceived maternal neglect was a significant early predictor of mood disorders, even after adjusting for further factors, such as exposure to parental BPD. In addition, high offspring emotionality appeared to be associated with the development of mood disorders, also being the possible mediator of the relationship between maternal neglect and the development of mood disorders
Hillegers et al. (2004), The Netherlands, “The Dutch Bipolar Offspring Study”	I, II	–	12–21	DSM-IV K-SADS-PL FH-RDC K-LEDS FH-RDC Life event load (time-dependent variable)	Stressful life events	140 (49)	16.0 ± 2.7	–	–	Among offspring of parents with BPD, stressful life events increased the liability to mood disorders independently of the familial loading, but the effects slowly diminished over time

*BPD* bipolar disorder, *CBCL* Child Behavior Checklist for ages 6–18, *CBQ* Conflict Behavior Questionnaire, *CECA.Q* Childhood Experience of Care and Abuse Questionnaire, *CTQ* Child Trauma Questionnaire, *Dx* diagnosis, *EAS* Early Adolescence Temperament Scale, *EMBU* Swedish acronym for “my memories of upbringing”, *FACES-II* Family Adhesion and Cohesion Evaluation Scales-II, *FH-RDC* Family History Related Research Criteria, *HC* Healthy Controls, *K-LEDS* Kiddie Life Events and Difficulty Scale, *K-SADS-MRS* Kiddie Schedule for Affective Disorders and Schizophrenia, Mania Rating Scale, *K-SADS-PL* Kiddie Schedule for Affective Disorders and Schizophrenia, Present and Lifetime Version, *LEDS* Life Events and Difficulties Scale, *LEQ* Life Events and Difficulties Questionnaire, *Mood* any mood disorder, including bipolar disorder, *NR* not reported, *PDI* Parenting Dimensions Inventory, *QFP* Questionnaire for Family Problems, *TCI* Temperament and Character Inventory, *TRF* Teacher Report Form, *UCL* Utrecht Coping List, *w/o* without

Using data from a longitudinal high-risk study, we therefore aimed to characterize the role of a series of individual and familial environmental factors, reported by offspring prior to the onset of mood episodes, in the well-established parent–child transmission of BPD and MDD. More specifically, our goals were to (1) assess the association between parental BPD or MDD and individual and familial environmental factors including traumatic events in offspring, parental separation, family cohesion and parental attitudes, and (2) test whether these factors were mediators of the previously documented parent–child transmission of BPD and MDD. Given that, in accordance with previous research on early onset BPD (review Carlson and Pataki 2016), we had already documented the significant impact of an early age of onset of the parental BPD on the risk of BPD in offspring (Preisig et al. 2016), and given that research has also found a higher risk of MDD among offspring of parents with an early onset of MDD (Weissman et al. 1988), parental BPD and MDD were dichotomized in our analyses according to the age of onset.

## Methods

### Sample

**Fig. 1:**
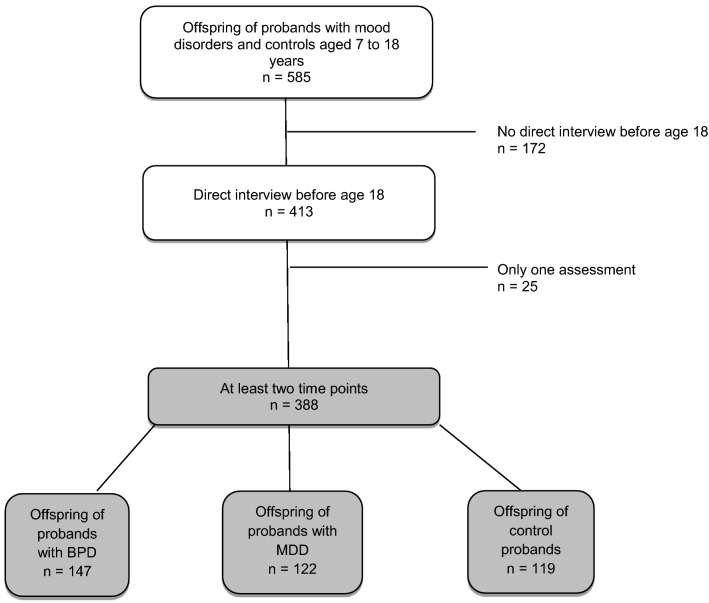
Flow chart of the sample selection of offspring of parents with mood disorders and controls. BPD bipolar disorders, MDD major depressive disorders

**Fig. 2:**
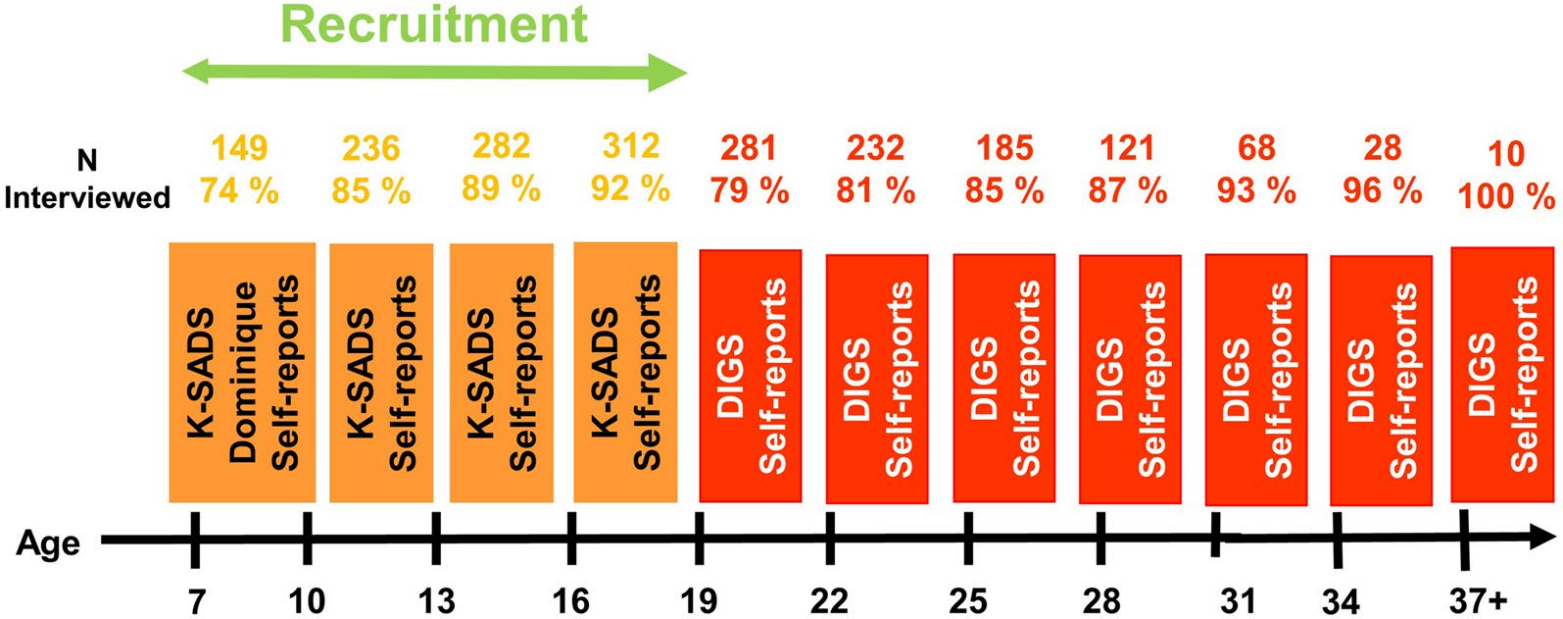
Assessments in offspring (n = 388). Mean follow-up length: 14 yrs (s.d. 4.6 yrs)

The sample stems from a large family study of mood disorders conducted in the French-speaking part of Switzerland (Vandeleur et al. 2014). We included the probands with participating offspring younger than 18 years at study intake in our offspring study. The methodology of this high-risk offspring study (named the Lausanne-Geneva cohort study of offspring of parents with mood disorders) has been described in detail (Vandeleur et al. 2017). Briefly, probands with mood disorders were consecutively recruited from the inpatient and outpatient facilities of the psychiatric departments of Lausanne and Geneva between 1996 and 2004. Inclusion criteria for probands with mood disorders were: (1) a lifetime diagnosis of bipolar-I, bipolar-II, schizoaffective bipolar disorder or MDD, and (2) having at least one participating biological child, aged 6.0 to 17.9 years at study intake. Inclusion criteria for the comparison probands, who were recruited from the orthopedic departments of Lausanne and Geneva, were: (1) the absence of a lifetime mood or psychotic disorder, and (2) the same inclusion criterion for offspring as that of the mood disorder cases. An effort was also made to interview all biological co-parents of offspring, a total of 123 co-parents having participated in direct interviews (64%). Parents and offspring were invited to take part in follow-up assessments at predetermined ages of the offspring (7, 10, 13, 16, 19, 22, 25, 28, 31, 34, 37 and 40 years). The selection of the study sample is depicted in Fig. 1. The description of the assessments at the predefined ages over the follow-up period is provided in Fig. 2.

Offspring who were included in our analyses and those who were not did not differ by sex, but those who participated were younger in age (9.8 vs. 11.3 years, b = − 1.4, 95% CI − 2.2; − 0.7, p < 0.001) and were less likely to present major depressive episodes (7.7% vs. 12.3%, OR = 2.0; 95% CI 1.1; 3.7, p < 0.05) according to the family history information provided by their parents. They did not differ regarding the likelihood of having any other psychiatric disorder.

### Procedures

#### Diagnostic assignment

Information on parents and adult offspring was obtained using the French version (Preisig et al. 1999) of the semi-structured Diagnostic Interview for Genetic Studies (DIGS) (Nurnberger et al. 1994) and offspring younger than 18 years were directly interviewed using a French translation of the Kiddie-Schedule for Affective Disorders and Schizophrenia-Epidemiologic version (K-SADS-E) (Orvaschel et al. 1982). In addition to direct interviews, information on children and parents was systematically elicited from all participants from the age of 15 years using the Family History-Research Diagnostic Criteria (FH-RDC) (Andreasen et al. 1977). The reliability of the French translation of the DIGS (Preisig et al. 1999; Berney et al. 2002), the reliability of the K-SADS-E (Orvaschel et al. 1982; Chambers et al. 1985; Vandeleur et al. 2012), and the validity of the French version of the FH-RDC (Rothen et al. 2009; Vandeleur et al. 2015) have been extensively tested. Interviewers were required to be at least bachelor-level psychologists and were trained over a 1- to 2-month period. They were blind to the disease status of the other family members. Each interview was reviewed by a senior psychologist to ensure data quality and accuracy.

Diagnoses were made over lifetime using a best-estimate procedure (Leckman et al. 1982), which relied on the combination of information from direct interviews, family history report(s), and medical records where available. Mood episodes and disorders were based on the DSM-5 whereas other mental disorders were diagnosed according to the DSM-IV. Age of onset in probands was based on the age of the first described full mood episode (mania, hypomania or major depressive episode (MDE) (Preisig et al. 2016).

#### Assessment of environmental factors

- Childhood trauma and familial factors assessed by direct interview
  As part of the post-traumatic stress disorder (PTSD) section of the K-SADS-E, the following childhood traumatic events were documented in offspring: victim of accident or severe catastrophe (car accident, other accident, fire, witness of a disaster), violent crime, sexual abuse and physical abuse or witnessing trauma to others (accident, violent crime, domestic violence). The variable was taken from the first assessment, if this assessment preceded the onset of the first mood episode. Similarly, the variable “parental separation” was derived from information of the first K-SADS interview. When a mood episode was already diagnosed at this assessment, parental separation status prior to this episode was established either from the child's first DIGS interview, which contains the date of parental separation, or from the parents' family history interviews that also collected information about with whom they lived at that time. The socio-economic status (SES) of the families was determined by the parental reports of the professional category and the level of education of each spouse of the household according to the Hollingshead Index (Hollingshead 1975).
- Familial environment factors assessed with self-reports
  Familial cohesion and the perception of maternal or paternal attitudes were assessed using the Family Adaptability and Cohesion Evaluation Scales version III (FACES III) (Olson et al. 1985) and the Parental Bonding Instrument (PBI) (Parker et al. 1979), respectively. These self-report questionnaires were completed in any assessment of offspring who were at least 10 years old. We only used data assessed prior to the onset of the first mood episode. Familial cohesion according to the FACES III is assessed by 10 questions. The Cronbach alpha of the French version of this scale was 0.78 (Vandeleur et al. 1999). The French version of the PBI revealed three factors in adolescents: “care”, “denial of autonomy” and “encouragement of freedom” (Tercier et al. 2011). The latter two factors partitioned the original “protection” factor into a negative pole (denial of autonomy) and a positive pole (encouragement of freedom). The Cronbach alphas were 0.88, 0.75 and 0.77 for the mother care, denial of psychological autonomy and encouragement of behavioral freedom factors, and 0.90, 0.74 and 0.77 for each of the father factors, respectively.

### Data analysis

Parental BPD and MDD were dichotomized in our analyses according to the age of onset (before vs. after the age 21 years) (Preisig et al. 2016). Univariate between-group analyses for categorical and continuous variables were performed using chi-square tests or ANOVA, respectively.

#### Association between parental early and later-onset BPD or early and later-onset MDD and individual and familial environmental factors

Associations between the proband’s mood disorder status and early trauma in offspring, parental separation, familial cohesion and parental attitudes were established using generalized linear mixed models (first study goal). These models were adjusted for sex and age of offspring*,* number of assessments in offspring, sex and age of probands, SES of the family, proband non-mood disorders (anxiety disorders, alcohol/ drug, abuse/dependence) and spouse mood (bipolar and unipolar) and non-mood disorders, as well as intra-familial correlations (varying number of offspring across families).

#### Mediation analysis

We tested whether the individual or familial environmental factors were mediators of the association between exposure to parental mood disorders and the onset of mood disorders in offspring according to the four criteria postulated by Baron and Kenny (1986) and the definitions of MacKinnon, Krull and Lockwood (MacKinnon et al. 2000). According to these four criteria, mediation exists if: (1) independent (exposure to parental mood disorder) and dependent variables (onset of mood disorders in offspring) are associated; (2) independent variable and mediator (individual or familial environmental factors) are associated (first study goal); (3) mediator and dependent variable are associated; and (4) after adjustment for the effect of the mediator, mediator and dependent variable remain significantly associated but independent and dependent variables are either no longer associated (full mediation), or still associated but reduced in strength (partial mediation).

The associations between the individual or familial environmental factors and the onset of BPD or MDD in offspring were tested in one overall model for each outcome, using Cox regression models (Therneau et al. 2003), adjusting for sex of offspring, number of assessments in offspring as well as for SES and intra-familial correlations (3rd criterion of Baron and Kenny).

The prospective associations between proband mood disorder status and the onset of (hypo)manic episodes or MDD in the offspring, before (1st criterion of Baron and Kenny) and after adjustment for potential mediators (4th criterion of Baron and Kenny), were established using Cox regression models (Therneau et al. 2003). Potential mediators were tested if the previous analysis had shown them to be associated with both parental mood disorder status and the risk of mood disorders in offspring at the lenient *p* < 0.1 level of significance. These models were adjusted for sex of offspring, number of assessments in offspring, sex and age of probands, SES of the family, proband non-mood disorders, spouse mood and non-mood disorders, and intra-familial correlations.

In models using diagnostic data in co-parents, 100 multiple imputations were performed using the MissForest procedure based on random forests (Stekhoven and Buhlmann 2012) for missing data (n = 21). Similarly for models using data from self-reports as independent variables, missing data on parental separation (n = 9), the FACES III, PBI proband or PBI spouse (42–45% missing data depending on the subscale) were imputed. To control for consistency of results, these models were repeated using a reduced sample without any imputations.

All analyses were conducted using the Statistical Analysis System, version 9.4 (SAS Institute, Inc., Cary, NC, USA), and the statistical analyses environment R (R Core Team. R Foundation for Statistical Computing, Vienna, Austria. http://www.R-project.org/).

## Results

### Description of the cohort

The average number of assessments of the 388 offspring 5.1 (s.d. = 1.6; range: 2–9) with a mean duration of 14.0 years of follow-up (s.d.: 4.6). More than 80% of the offspring assessments relied on direct interviews. The mean offspring age at the first and last assessment was 9.9 years (s.d. = 4.4 years) and 23.9 years (s.d. = 6.5 years), respectively. Table 2 shows the characteristics of probands, spouses and offspring as a function of the proband's mood disorder status. Probands differed in terms of age, SES, lifetime history of anxiety and alcohol use disorders. The spouses grouped according to the proband’s mood disorder status only differed by lifetime alcohol use disorders, whereas offspring differed by the age of the last assessment, number of assessments or interviews, and follow-up duration.

**Table 2:** Sample characteristics

Probands (N = 214)	BPD onset < 21 yrs (n = 30)	BPD onset > 21 yrs (n = 51)	MDD onset < 21 yrs (n = 21)	MDD onset > 21 yrs (n = 47)	Comparison (n = 65)	Statistic	p
Socio-demographic factors							
Age (yrs), mean (s.d.)	38.3 (7.3)	41.5 (6.0)	37.9 (5.5)	42.4 (7.8)	41.1 (6.8)	F_4_ = 2.7	0.032
Female, %	60.0	54.9	71.4	53.2	43.1	χ^2^_4_ = 6.1	n.s
Socio-economic status, mean (s.d.)^a^	3.0 (1.1)	3.3 (1.0)	2.8 (1.1)	2.8 (1.0)	3.4 (1.1)	F_4_ = 2.8	0.025
Non-mood disorders at baseline, %							
Any anxiety disorders^b^	30.0	29.4	47.6	42.6	6.2	χ^2^_4_ = 24.9	< 0.001
Alcohol abuse or dependence	26.7	27.5	33.3	51.1	9.2	χ^2^_4_ = 24.3	< 0.001
Illicit substance abuse or dependence	23.3	17.7	9.5	19.2	7.7	χ^2^_4_ = 5.8	n.s

Spouses (N = 193)	Spouses of probands with BPD onset < 21 yrs (n = 27)	Spouses of probands with BPD onset > 21 yrs (n = 46)	Spouses of probands with MDD onset < 21 yrs (n = 17)	Spouses of probands with MDD onset > 21 yrs (n = 42)	Spouses of comparison probands (n = 61)		
Socio-demographic factors							
Age (yrs), mean (s.d.)	40.8 (9.0)	43.1 (7.7)	44.3 (8.0)	42.4 (6.2)	43.1 (7.9)	F_4_ = 0.7	n.s
Female^c^, %	40.0	45.1	28.6	46.8	56.9	χ^2^_4_ = 6.1	n.s
Interviewed^c^ (%)	50.0	60.8	42.9	53.2	66.2	χ^2^_4_ = 5.1	n.s
Spouse’s disorders at baseline, %							
Any bipolar disorder	11.1	6.5	5.9	9.5	1.6	χ^2^_4_ = 4.1	n.s
Any depressive disorder	22.2	34.8	41.2	28.6	29.5	χ^2^_4_ = 2.3	n.s
Any anxiety disorders^b^	29.6	10.9	17.7	23.8	14.8	χ^2^_4_ = 5.4	n.s
Alcohol abuse or dependence	18.5	23.9	35.3	23.8	6.6	χ^2^_4_ = 10.6	< 0.05
Illicit substance abuse or dependence	7.4	4.4	0	11.9	3.3	χ^2^_4_ = 5.0	n.s

Offspring (N = 388)	Offspring of probands with BPD onset < 21 yrs (n = 52)	Offspring of probands with BPD onset > 21 yrs (n = 95)	Offspring of probands with MDD onset < 21 yrs (n = 40)	Offspring of probands with MDD onset > 21 yrs (n = 82)	Offspring of comparison probands (n = 119)		
Socio-demographic factors							
Age at first follow-up (yrs), mean (s.d.)	8.6 (5.1)	10.9 (4.1)	8.8 (3.4)	10.7 (3.9)	9.3 (4.8)	F_4_ = 2.2	n.s
Age at last follow-up (yrs), mean (s.d.)	21.6 (7.3)	26.7 (5.6)	21.7 (4.4)	23.5 (6.4)	23.6 (6.8)	F_4_ = 4.0	0.004
Girls, %	61.5	47.4	67.5	45.1	46.2	χ^2^_4_ = 9.4	n.s
Number of assessments (%)	4.9 (1.7)	5.6 (1.6)	4.7 (1.1)	4.7 (1.6)	5.4 (1.6)	F_4_ = 6.0	< 0.001
Number of direct interviews (%)	3.8 (1.8)	4.8 (1.9)	3.9 (1.4)	3.8 (1.8)	4.2 (1.9)	F_4_ = 4.3	< 0.01
Duration of follow-up [yrs], mean (s.d.)	13.0 (5.2)	15.8 (4.5)	12.9 (3.3)	12.8 (4.1)	14.3 (4.7)	F_4_ = 6.7	< 0.001

*BPD* bipolar disorder, *MDD* major depressive disorder, *yrs* years, *sd* standard deviation, *n.s.* not statistically significant

^a^A value of 3 represents an SES of III (middle class) on the Hollingshead Scale

^b^Includes generalized anxiety disorder, social phobia, panic disorder, or agoraphobia

^c^This information was derived for 21 spouses with otherwise missing data

### Associations between proband mood disorder status and individual/familial environmental factors

Among all offspring, 21.4% had experienced one or more traumatic events of which 12.6% were accidents, 4.4% were violent crime, 3.4% were sexual abuse, 4.6% were physical abuse and 18.0% were witnessing domestic violence or accidents to others. Almost half of these offspring (45.8%) had experienced two or more of these events until the last time of reporting. Table 3 shows that offspring of probands with later onset BPD and offspring of probands with early-onset and later onset MDD reported having had traumatic events more frequently than offspring of comparison probands. Offspring of all groups of probands with mood disorders had also been exposed to parental separation more frequently than offspring of controls. Additionally, offspring of probands with early onset BPD scored lower on family cohesion. Regarding parental attitudes, lower parental care was reported by offspring for both probands with early onset BPD and their spouses compared to offspring of controls. Offspring of probands with later onset BPD also reported lower care for co-parents. Interestingly, these offspring reported lower and not higher levels of denial of autonomy for both of their parents. In addition, a trend was found for lower family cohesion scores in offspring of probands with later onset MDD compared to offspring of controls.

**Table 3:** Individual or familial environmental factors according to offspring by proband mood disorder status

	Proband diagnostic status								
	BPD onset < 21 yrs		BPD onset > 21 yrs		MDD onset < 21 yrs		MDD onset > 21 yrs		Comparison
	%/m (SD)	OR^a^ or β^a^ (95% CI)	%/m (SD)	OR^a^ or β^a^ (95% CI)	%/m (SD)	OR^a^ or β^a^ (95% CI)	%/m (SD)	OR^a^ or β^a^ (95% CI)	%/m (SD)
Childhood adversity									
Traumatic events (N = 388)	21.2	OR = 2.0 (0.7, 5.4)	28.4	OR = 2.5* (1.1, 5.5)	30.0	OR = 3.2* (1.1, 9.4)	24.4	OR = 2.6* (1.0, 6.8)	10.9
Family environment									
Parental separation (N = 388)	69.2	OR = 3.1** (1.4, 6.9)	76.8	OR = 5.2*** (2.6, 10.3)	75.0	OR = 3.5* (1.3, 9.1)	67.1	OR = 3.5** (1.7, 7.4)	40.3
Family cohesion (N = 224)	31.4 (8.7)	β = − 5.3** (− 8.9, − 1.7)	35.2 (7.4)	β = − 0.8 (− 3.3, 1.8)	34.3 (7.1)	β = − 0.0 (− 4.1, 4.1)	32.8 (7.8)	β = − 3.1° (− 6.3, 0.2)	36.3 (7.6)
Parental attitudes									
Proband (N = 221)									
Care	25.7 (7.5)	β = − 5.0*** (− 7.7, − 2.4)	28.8 (6.2)	β = − 1.3 (− 3.3,0.7)	29.4 (4.3)	β = − 0.9 (− 4.1,2.2)	28.4 (6.4)	β = − 1.6 (− 4.1,0.9)	29.8 (5.2)
Denial of autonomy	6.1 (4.5)	β = 1.0 (− 0.9, 2.8)	3.9 (3.2)	β = − 1.4* (− 2.7, − 0.1)	5.8 (3.2)	β = 0.0 (− 2.1, 2.1)	5.7 (4.4)	β = − 0.3 (− 2.0, 1.4)	5.4 (3.9)
Encouragement of freedom	12.2 (4.0)	β = − 0.4 (− 2.0, 1.3)	12.9 (3.2)	β = 0.6 (− 0.6, 1.8)	13.3 (3.2)	β = 0.7 (− 1.2, 2.6)	12.6 (3.9)	β = 0.3 (− 1.2, 1.8)	12.1 (3.6)
Spouse (N = 223)									
Care	26.3 (8.4)	β = − 4.1** (− 7.2, − 1.0)	26.9 (6.6)	β = − 2.7* (− 5.0, − 0.4)	29.5 (5.7)	β = − 0.6 (− 4.2, 3.0)	28.6 (6.3)	β = − 0.8 (− 3.7, 2.1)	29.3 (6.6)
Denial of autonomy	5.1 (3.5)	β = − 0.4 (− 2.2, 1.4)	3.9 (3.4)	β = − 1.6* (− 3.0, − 0.3)	6.4 (4.5)	β = 1.3 (− 0.8, 3.4)	5.0 (4.1)	β = − 0.3 (− 2.0, 1.4)	6.0 (4.1)
Encouragement of freedom	12.5 (4.2)	β = − 0.2 (− 1.7, 1.4)	12.3 (3.4)	β = − 0.4 (− 1.5, 0.8)	12.0 (4.0)	β = − 1.0 (− 2.8, 0.8)	13.7 (3.0)	β = 1.2 (− 0.2, 2.7)	12.2 (3.1)

Statistically significant values are in bold

*BPD* bipolar disorder, *MDD* major depressive disorder, *yrs* years, *m* mean score, *SD* standard deviation, *OR* odd ratio, *β* beta estimate, *95% CI* 95% confidence intervals

^a^Models adjusted for sex, age and number of assessments in offspring, sex and age in proband, socio-economic status of the family, proband non-mood disorders and spouse mood and non-mood disorders (one single model for each outcome variable, imputed for missing spouse disorders)

***p < 0.001

**p < 0.01

*p < 0.05

°*p* < 0.1

### Associations between exposure to individual/familial environmental factors and the onset of (hypo)manic episodes or MDD in offspring

According to Table 4 only childhood trauma was a significant predictor of MDD in offspring, whereas parental separation was a predictor of mania/hypomania in offspring, on the trend level. The results did not change when analyses were restricted to offspring with unimputed data (results not shown).

**Table 4:** Onset of mood episodes or disorders in offspring by preceding individual or familial environmental factors

	Mania/hypomania onset in offspring			MDD onset in offspring^b^		
	Yes	No	HR^a^ (95% CI)	Yes	No	HR^a^ (95% CI)
	%/m (SD)	%/m (SD)		%/m (SD)	%/m (SD)	
	N = 42	N = 346		N = 181	N = 165	
Childhood adversity						
Traumatic events	33.3	19.9	1.3 (0.7, 2.7)	35.4	3.0	2.8*** (2.0, 3.8)
Family environment						
Parental separation	78.6	60.4	2.2° (1.0, 4.7)	65.2	55.2	1.1 (0.8, 1.6)
Family cohesion	34.8 (5.0)	34.7 (7.9)	1.0 (0.9, 1.1)	35.1 (8.1)	34.5 (7.9)	1.0 (1.0, 1.0)
Parental attitudes						
Proband						
Care	28.5 (5.2)	28.9 (6.0)	1.0 (0.6, 1.5)	28.9 (6.1)	28.8 (6.0)	1.0 (0.8, 1.2)
Denial of autonomy	4.3 (3.2)	5.2 (3.9)	1.0 (0.6, 1.5)	5.1 (3.7)	5.4 (4.1)	1.0 (0.8, 1.3)
Encouragement of freedom	13.6 (2.3)	12.4 (3.6)	1.0 (0.6, 1.5)	12.9 (3.8)	12.2 (3.5)	1.0 (0.8, 1.3)
Spouse						
Care	27.6 (4.3)	28.3 (6.9)	1.1 (0.7, 1.7)	28.2 (6.9)	28.4 (6.9)	1.0 (0.8, 1.3)
Denial of autonomy	5.2 (3.4)	5.2 (4.0)	1.1 (0.7, 1.6)	5.2 (4.1)	5.3 (4.0)	1.1 (0.8, 1.3)
Encouragement of freedom	13.4 (2.9)	12.4 (3.4)	1.1 (0.7, 1.6)	12.8 (3.2)	12.2 (3.6)	1.0 (0.8, 1.3)

Statistically significant values are in bold

*MDD* major depressive disorder, *m* mean value, *SD* standard deviation, *HR* hazard ratios, *95% CI* 95% confidence intervals

***p < 0.001

**p < 0.01

°*p* < 0.1

^a^One overall model for the two offspring outcomes with imputations for mediating variables, adjusted for sex and number of assessments in offspring, and SES of the family

^b^Excluding offspring with mania/hypomania

### Associations between proband mood disorder status and onset of (hypo)manic episodes or MDD in the offspring before and after adjustment for potential mediators

According to the previous analyses, only two variables met predefined criteria for potential mediators: parental separation for the association between parental BPD and the onset of (hypo)manic episodes in offspring and traumatic events for the association between parental MDD and the emergence of this disorder in offspring. Table 5 reveals that the HR for the strong association between early-onset parental BPD and the emergence of (hypo)manic episodes in offspring (Model 1) only diminished of 15.0% from 8.0 to 6.8 after the introduction of the effect of parental separation and remained highly significant, whereas parental separation was not significantly associated with the emergence of (hypo)manic episodes in offspring (Model 2). Similarly, the HR for the initially significant association between early-onset MDD in parents and MDD in offspring (Model 1) decreased of 21.1% from 1.9 to 1.5 after the introduction of the effect of traumatic events into the model for MDD (Model 2). Despite this rather modest decrease of the HR for the association between early-onset MDD in parents and MDD in offspring, this association failed to reach the level of statistical significance after the introduction of the effect of traumatic events, whereas traumatic events remained significantly associated with the risk of MDD in offspring.

**Table 5:** Onset of episodes/disorders in offspring by proband status with or without adjustment for potential mediators

	Mania/hypomania onset in offspring				MDD onset in offspring^c^			
	Model 1^a^		Model 2^b^		Model 1^a^		Model 2^b^	
	HR (95% CI)	*p*	HR (95% CI)	*p*	HR (95% CI)	*p*	HR (95% CI)	*p*
Parental mood disorder								
BPD onset < 21 yrs	**8.0 (3.1–20.7)**	**< 0.001**	**6.8 (2.6–18.1)**	**0.001**	1.1 (0.6–2.0)	0.790	1.0 (0.6–1.9)	0.941
BPD onset > 21 yrs	1.1 (0.4–3.0)	0.930	0.9 (0.3–2.6)	0.824	0.9 (0.6–1.4)	0.710	0.8 (0.5–1.3)	0.366
MDD onset < 21 yrs	1.4 (0.3–5.6)	0.668	1.2 (0.3–4.8)	0.843	**1.9 (1.1–3.2)**	**0.026**	1.5 (0.8–2.6)	0.191
MDD onset > 21 yrs	0.6 (0.2–2.5)	0.514	0.5 (0.1–2.2)	0.384	1.3 (0.8–2.1)	0.402	1.1 (0.6–1.8)	0.783
Potential mediators								
Traumatic events	–	–	–	–	–	–	**2.5 (1.7–3.6)**	**< 0.001**
Parental separation	–	–	1.8 (0.8–4.2)	0.160	–	–	–	–

Statistically significant values are in bold

*BPD* bipolar disorder, *MDD* major depressive disorder, *HR* hazard ratios, *95% CI* 95% confidence intervals

^a^Model 1 (imputed) with no mediators, adjusted for sex, age and number of assessments in offspring, sex and age in proband, socio-economic status of the family, proband alternate and non-mood disorders, spouse mood and non-mood disorders and intra-familial correlations

^b^Models (imputed) successively including potential mediators, adjusted for the same variables as Model 1

^c^Offspring with mania/hypomania excluded

## Discussion

Using data from a controlled prospective high-risk study including offspring of parents with BPD and MDD, this is the first paper to simultaneously test the role of a series of adverse environmental factors, reported by offspring still exempt of mood disorders, on the well -established parent–child transmission of mood disorders including information from both parents. The most salient findings of the current study were that (1) offspring of parents with BPD or MDD reported several adverse environmental factors more frequently than offspring of controls, (2) these factors did not account for the strong association between parental BPD with early onset and the elevated risk of (hypo)manic episodes in offspring, whereas there was evidence for partial mediation of the association between parental MDD with early onset and the elevated risk of MDD in offspring via traumatic events.

### Associations between proband mood disorder status and individual/familial environmental factors

The associations observed between mood disorders in parents and an elevated frequency of childhood trauma and parental separation in their offspring as well as low family cohesion and unfavorable parental attitudes in these families are consistent with findings from previous research. Indeed, childhood trauma has been frequently documented in families of patients with BPD (Aas et al. 2016) and in families of mothers with MDD (Najman et al. 2017), whereas parental separation was frequently observed in families with parental depression (Beardslee et al. 2011). Moreover, parental BPD has consistently been found to be associated with lower parent-reported cohesion when compared to parents without psychiatric disorders (Stapp et al. 2020a). Similarly, families of parents affected by mood disorders revealed poor parental rearing, reported by parents (Iacono et al. 2017) and offspring (Lau et al. 2018). Our results are essentially in line with these previous findings. Indeed, except for the children of patients with early-onset BPD, offspring of patients with mood disorders were more likely to report childhood adversity than offspring of controls at least on the trend level. Similarly, the offspring of all four groups of affected parents experienced parental separation more frequently than offspring of controls. In addition, offspring of parents with early onset BPD reported lower family cohesion and lower parental care compared to offspring of controls. The offspring of parents with later onset BPD also reported a lower level of care from the co-parent, but also lower levels of denial of psychological autonomy from both parents—an indicator of favorable parental attitudes.

### Mediators of the association between BPD in parents and the risk of (hypo)manic episodes in offspring

Our study revealed a strong parent–child transmission of BPD, which was restricted to the families of patients with early-onset BPD. However, despite more frequent reporting of traumatic events and unfavorable family characteristics by offspring of parents with BPD, our data did not support a significant mediation of this parent–child transmission via these factors. This was due to the fact that none of the factors associated with having a parent with BPD was significantly associated with the subsequent development of (hypo)manic episodes in offspring. Although parental separation was associated with the emergence of (hypo)manic episodes in offspring on a trend level, the inclusion of this variable in the model only modestly diminished the HR for the association between parental early-onset BPD and the emergence of (hypo)manic episodes in offspring, which remained highly significant.

Our data suggesting that prospectively assessed traumatic events are not a risk factor for the subsequent onset of BPD in offspring of parents with BPD, are consistent with the results of the Pittsburgh Bipolar Offspring Study (BIOS), in which a history of physical and/or sexual abuse was not associated with the development of offspring BPD among high-risk offspring (Goldstein et al. 2010). However, these findings contrasts with those of other previous studies (Menculini et al. 2020). Recently, the Dutch Bipolar Offspring Study showed that emotional maltreatment from parents reported by the offspring was associated with the development of mood disorders among high-risk offspring (Koenders et al. 2020). However, adversity was reported retrospectively by the offspring after the onset of mood psychopathology, which may have introduced reporting bias. Aas and colleagues (Aas et al. 2016) pointed out in their review of studies on adults with BPD that childhood trauma could be a salient risk factor for the future development of BPD. Possible explanations for the discrepant findings between studies of offspring at high-risk and those of patients with BPD are: (1) the occurrence of adverse traumatic events may be more critical for the development of BPD in patients who are not necessarily born in high-risk families than in offspring who have parents with BPD, and (2) adult patients with BPD may be more likely to retrospectively report life stress or subsequent trauma than adults who do not have BPD (recall bias) (Frissa et al. 2016). The fact that the other measured family environmental factors were not associated with the risk of (hypo)manic episodes in offspring in our study is in line with findings of the Dutch high-risk study showing that family functioning assessments completed by parents with BPD were not associated with the subsequent development of mood disorders in offspring (Koenders et al. 2020). This was confirmed by a systematic review on the topic (Menculini et al. 2020).

The absence of significant prospective associations between the measured environmental factors and the risk of (hypo)manic episodes in offspring in our study as well as the absence of significant mediation of the association between parental early-onset BPD and the risk of (hypo)manic episodes in offspring by these factors is compatible with the hypothesis that mostly genetic factors account for the parent–child transmission of BPD. This conclusion is in line with the results of a recent a large-scale Swedish national registry study analyzing high-risk parent–offspring and adoption constellations (Kendler et al. 2020a).

### Mediators of the parent–child transmission of MDD

Regarding the parent–child transmission of MDD, we could replicate a significant association between parental MDD with early onset and the risk of this disorder in children, which is consistent with those of previous high risk studies (Weissman et al. 1988; Rice et al. 2019). Among the tested environmental factors, exposure to traumatic events was more frequently reported in the children of parents with MDD compared to those of controls. Exposure to these events was also significantly associated with the subsequent risk of developing MDD in offspring. Although the inclusion of this variable only modestly diminished the size of the association between parental early-onset MDD and the risk of MDD in offspring, this association shortly failed to reach the level of statistical significance which, according to the pre-defined criteria, supports mediation. Hence, the parent–child transmission of MDD could be partially explained by the more frequent occurrence of traumatic events in offspring of parents with early-onset MDD implying a higher risk for the subsequent development of MDD in children. This finding is consistent with previous prospective studies that showed both adverse life events to occur more frequently in families with maternal MDD (Najman et al. 2017) and to increase the risk for MDD in offspring (Najman et al. 2017; Asselmann et al. 2018). Other studies found poor parent–child relationships combined with childhood maltreatment by age 11 to predict the onset of MDD among offspring from the community (Wilson et al. 2014) or traumatic events and separation from parents combined with parental symptoms of depression to predict the incidence of mood psychopathology in offspring (Zimmermann et al. 2008).

Moreover, the Swedish national registry study also suggested that both parental MDD and a disrupted family environment in terms of parental death or divorce had a meaningful impact on the risk of MDD in offspring (Kendler et al. 2020b).

### Limitations of the study

The results of this study should also be interpreted in the context of several limitations. First, the relatively small sample size of offspring that developed mania/hypomania limited the statistical power of analyses with this outcome. For this reason, we applied a less stringent threshold (*p* < 0.1) for the identification of environmental factors that could play a role in the parent–child transmission of mood disorders. Second, we needed to impute about a third of data on familial cohesion and parental attitudes due to incomplete filling in of self-report instruments across all the study waves. However, comparison between imputed and non-imputed data did not reveal evidence of bias. Third, the wide age range of the offspring at inclusion into the study may have introduced heterogeneity into our findings. Fourth, despite the prospective design of our study, the information collected for the 3-year interval of time between evaluations was necessarily retrospective. Fifth, our testing of potential mediators did not include all environmental characteristics; factors that were not measured such as parent–child conflicts may be associated with the risk of BPD emergence in offspring in high-risk families (Stapp et al. 2020a). Sixth, as a part of the co-parent diagnoses relied on information from family history reports, it was not possible to further delineate co-parental mood diagnoses according to their ages of onset.

### Conclusions

The current prospective study including diagnostic information on both parents, a long follow-up and the restriction to only premorbid measurements of the individual and family environment addresses many of the limitations of previous research, and shows that the strong association between early-onset parental BPD and the elevated risk of (hypo)manic episodes in offspring is not mediated by the adverse environmental factors measured in our study. In contrast, childhood trauma modestly mediates the association between parental early-onset MDD and the increased risk of MDD in offspring. Among the assessed traumatic events, only a part might be preventable by therapeutic measures (e.g. sexual or physical abuse, witnessing of familial violence). However, early therapeutic efforts in offspring exposed to these events are likely to reduce their deleterious impact on the risk of subsequent MDD.

## Data Availability

The datasets used and/or analyzed during the current study are available from the corresponding author on reasonable request.
